# Mass spectrometry measurements of mercury isotope ratios support geochemical sourcing of archaeological cinnabar in the Andean region

**DOI:** 10.1371/journal.pone.0326414

**Published:** 2025-07-07

**Authors:** Michelle Young, Colin Cooke, Richard Burger, Emily Kaplan, Gabriel Prieto, Jacob Bongers, Jordan Dalton, Fathima Usama, Shengliu Yuan, Holger Hintelmann

**Affiliations:** 1 Department of Anthropology, Vanderbilt University, Nashville, Tennessee, United States of America; 2 Department of Earth and Atmospheric Sciences, University of Alberta, Edmonton, Canada; 3 Department of Anthropology, Yale University, New Haven, Connecticut, United States of America; 4 Smithsonian Institution National Museum of the American Indian, Washington, DC, United States of America; 5 Department of Anthropology, University of Florida, Gainesville, Florida, United States of America; 6 Discipline of Archaeology, University of Sydney, Camperdown, Australia; 7 Department of Anthropology, SUNY Oswego, Oswego, New York, United States of America; 8 Water Quality Centre, Trent University, Peterborough, Ontario, Canada; 9 School of Ocean and Environment, Tianjin University of Science & Technology, Tianjin, China; Universidad de Sevilla, SPAIN

## Abstract

Geochemical methods can identify the long-distance exchange of resources in the archaeological record. Cinnabar is a mineral with a limited number of geological sources; however, methods for determining the geological origin of cinnabar are constricted by the limited availability of comparative geological source materials. This study applies a multi-method approach to compare isotopic ratios of mercury and sulfur in archaeological specimens of cinnabar from museum collections and scientifically excavated materials from the Andes region of South America. We demonstrate that the δ^202^Hg to Δ^199^Hg relationship, assessed through Multicollector Inductively Coupled Plasma Mass Spectrometry (MC-ICP-MS), falls along a predictive slope, while Isotope Ratio Mass Spectrometry (IR-MS) for sulfur (S) was not a reliable proxy for determining ore source. Furthermore, Hg isotope ratios from similar sites and contexts tended to cluster, suggesting that most sites exploited cinnabar from the same ore source. Statistical analyses support the idea that the Huancavelica deposit served as the primary source of cinnabar pigment for pre-Hispanic societies, while also revealing some intriguing divergences that suggest alternate sources were exploited during certain periods on the North and South Coasts of Peru. These results demonstrate that MC-ICP-MS analyses of mercury can be used to geochemically trace cinnabar ore in the Andes and beyond.

## Introduction

Decorative and symbolic use of red pigment has been a defining feature of modern human behavior for at least the past 50,000 years [1] and may even constitute the earliest symbolic tradition exhibited by our species [2]. In the pre-Hispanic record of the Central Andes, red colorant has played a central role in the materialization of Indigenous belief systems for millennia [3–8]. Material studies of red pigments offer insights into ancient acquisition strategies, exchange networks, craft production methods, and value systems. Growing interest in pigments of the ancient Andes and development of non-invasive and minimally invasive analytical techniques have generated a surge in chemical identification studies, which have revealed a range of mineral substances used in the creation of red colorants [9–22].

Despite recent advances in the identification of red pigments, determining the geological source of these minerals is a comparatively recent frontier in the archaeological sciences. This paper presents an important advance in geochemical sourcing of cinnabar pigment through the analysis of stable isotopes of mercury (Hg). Building upon a previously developed protocol and reference samples [11], this study includes a total of 97 pigment specimens, recovered from well-documented archaeological excavations and sampled from objects in museum collections. The stable isotopes of mercury were analyzed with Multicollector Inductively Coupled Plasma Mass Spectrometry (MC-ICP-MS). Isotope Ratio Mass Spectrometry (IR-MS) analyses of sulfur isotopes were also conducted on an additional subset of four (n = 4) samples to test the feasibility of using the sulfur isotope system as a complementary analytical method. The results of these analyses demonstrate that mercury’s isotopic system offers a promising method to determine the geological source(s) of cinnabar ore while more caution must be exercised when interpreting sulfur isotope data.

### Andean cinnabar

Cinnabar, or mercury sulfide (HgS), is a globally occurring, hydrothermal mineral that typically forms through precipitation, producing veins and pockets in fissures of sandstone, limestone, or volcanic rock [23]. Natural cinnabar is often found in gold/silver/arsenic/antimony deposits and is frequently associated with stibnite; it may also appear in association with pyrite, sphalerite, chalcopyrite, galena, arsenopyrite, realgar, marcasite, orpiment, tetrahedrite, quartz, barite, calcite and other Hg-, Sb- and Pb-based phases [24]. Cinnabar mineral exhibits a range of red colors dependent on the deposit and its conditions for crystallization. While cinnabar may result from a primary formation through which the cinnabar forms at the same time as the rock, secondary formation can also occur, through which mercury-rich hydrothermal fluids encounter sulfur-rich minerals in the host rocks.

**Fig 1:**
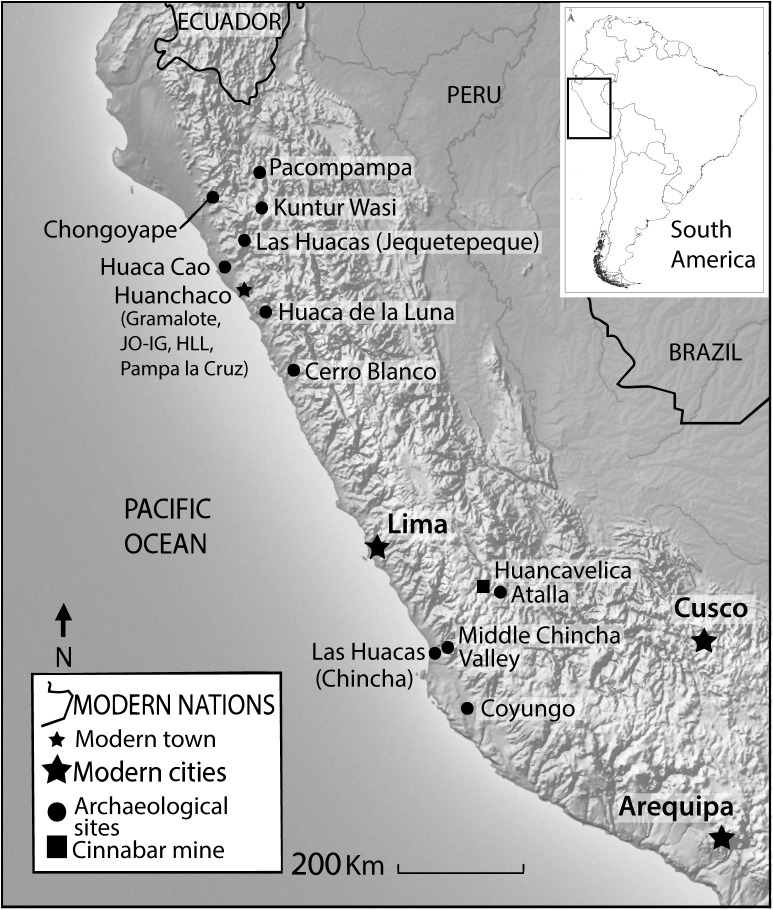
Map of archaeological sites mentioned in the text in reference to the Huancavelica cinnabar deposit. Map was created by Céline Erauw and Michelle Young. Topographic imagery was made with Natural Earth. South American Outline Map was sourced from GADM: https://gadm.org/license.html.

Cinnabar, also known as vermillion, or *illimpi* to the Inca, was a red colorant of particular importance for ancient Andean societies. Jesuit missionaries attest that native peoples continued to use this mercury-based mineral for decorating objects and their own bodies into the 16^th^ and 17^th^ centuries CE [25,26] (see also [27] for review of ethnohistoric evidence for cinnabar’s used in body painting and cosmetics among the Inca) but the use of this precious material was used for millennia prior to Spanish arrival in the Americas. Cinnabar appears as a powdered mineral pigment found in the archaeological record of the Andes in burial contexts and on finely crafted ritual objects [28–34]. The earliest chemically confirmed use of cinnabar pigment in the Andean region is from the site of Pampa Gramalote (Gramalote), a small fishing village on the far north coast of Peru where Prieto and colleagues [19] recorded intensive use beginning between 1500 and 1200 BCE. Their preliminary MC-ICP-MS analysis demonstrated consistency between the isotopic signature of ore from the Huancavelica source and the samples from Gramalote, leading investigators to suggest the cinnabar originated from the Huancavelica region, some 730 km away (Fig 1). Kriss and colleagues’ [16] study of the pigments used in the production of Paracas ceramic vessels from the south coast of Peru also noted that cinnabar pigments were employed to produce reds on Early Paracas vessels dating to the early first millennium BCE but were replaced by iron oxide-based reds in later periods.

These archaeological findings of Andean cinnabar pigments provide several key ideas that guide the current study. Firstly, since the 2^nd^ millennium BCE, ancient Andean systems of acquisition and exchange were far-reaching, connecting distant coastal societies to the Huancavelica cinnabar source at the Santa Barbara mine [11,19,35,36]. However, these networks fluctuated through time, and there was more than one natural cinnabar source that was exploited by Indigenous peoples prior to Spanish arrival to South America. This research tests a developing methodology to provide insights into the geological sources of archaeological cinnabar. Elucidating such temporal and geographic patterns of cinnabar distribution across Andean communities will allow us to map out shifting exploitation strategies as well as political and economic relationships between Indigenous groups dating far back into pre-Hispanic times.

### Application of stable isotopes in geochemical sourcing of archaeological cinnabar

Cinnabar sourcing studies that attempt to match stable isotope ratios of lead, sulfur and mercury to archaeological specimens have appeared sporadically in the literature over the last couple of decades. In addition to investigations of archaeological cinnabar from the Andean region [3,4,11,19,27,35–37], global studies have primarily been produced by teams working on the Iberian Peninsula during the Neolithic and Chalcolithic periods [38–41], Japanese tombs [42–44], and Roman wall paintings [45–48].

Lead is a trace element in cinnabar deposits, and despite the potential for interference between mercury and lead isotopes (²º⁴Hg and ²º⁴Pb), lead is the most frequently used isotope system for determining cinnabar ore provenance. Stable isotopes of lead (²º⁴Pb, ²º⁶Pb, ²º⁷Pb and ²º⁸Pb) have been used successfully to discriminate between several cinnabar ore deposits in the Iberian Peninsula [38,39] and they have been used in the Andes for geological attribution of metals [49,50]. As with geochemical sourcing studies of any material, the combination of cinnabar from different sources could complicate sourcing results [51] and lead isotopes alone may not be sufficient to pinpoint the origin of ore used in the production of any specific object [52].

Stable isotopes of sulfur have also been explored by previous investigators as a potential means to distinguish between cinnabar mine sources, with limited success. Sulfur isotopes were shown to differentiate broadly between Japanese and Chinese cinnabar deposits but the use of sulfur isotopes to distinguish between individual mine sources for cinnabar presents a challenge [42–44]. In a study of Roman wall paintings, investigators compared δ³⁴S detected in cinnabar pigments to published values from known European mercury deposits [46]. They conclude that the cinnabar originated from the Almadén mine, despite the lack of overlap between δ³⁴S values and the range of the Almadén deposit (−11.8 to +8.8) [45,47]. For archaeological pigments in particular, the inclusion of sulfur from sources beyond cinnabar presents a potential complication, since archaeological pigments often include other sulfur-bearing compounds [36] and may also receive sulfur from organic sources. For this reason, in their investigation, Tsantini and colleagues [48] stressed the importance of multisampling. They highlight that alternate sources of sulfur such as gypsum (CaSO_4_) may alter the S isotope values and found overlap between sulfur ³⁴S/³²S ratios, both of which complicate interpretation of S isotope results [48].

Archaeological research has the capacity to reveal whether any steps in the pigment production process might have impacted the isotopic values relevant to geochemical sourcing. For example, Prieto and colleagues' [19] detailed study of red pigments from Gramalote demonstrated that residents ground down chunks of hematite and cinnabar into powders that were mixed with water and a greasy substance in shell containers to prepare the mineral colorants as paints. Bongers and colleagues’ [28] study of red pigments used in burials dating to the late pre-Hispanic through Colonial Period in the Chincha Valley of southern Peru offers additional insights into cinnabar pigment preparation. The distinctively “wet” appearance of pigment application on human remains led them to suggest that pigment processing as a multi-step transformation that used ground stone tools to pulverize the ore into a powder that was then mixed with a liquid such as water, or potentially an organic substance, although their analyses did not detect organic analytes. These studies did not explore analytical methods to evaluate whether heat treatment was applied as part of red pigment processing in the Andes, but it is well-established that ancient peoples heat-treated mineral pigments such as ochres to create different hues, such as in the thermal transformation of goethite into hematite, or to promote a softer texture that’s easier for grinding [53–56]. Given the possibility of thermal transformations, we must consider how heat may have formed part of the process of cinnabar pigment preparation.

Investigators interested in the use of isotope systems for interpretation of the archaeological record have employed experimental methods to investigate how cultural and technological processes may have impacted fractionation and subsequent isotopic signatures of materials. Eerkins and colleagues [57] found that the Fe isotopes in iron oxides were not affected by firing at high temperatures (up to 900°C) and were only minorly affected by the admixture of other materials, such as kaolinite or hematite, to the pigment. Their findings suggest that Fe isotopes remain largely identical to those present in the original ore material collected by ancient miners regardless of subsequent craft production processes such as heat treatment and reactions with other minerals. For Pb isotopes, studies of metal cupellation and lead glazes verified that ratios were impacted, but to such a minor degree that isotopic fractionation could be disregarded [58,59].

In the case of mercury-based minerals, calcination of mercury ore has been shown to produce shifts in fractionation relative to the primary ore signatures inherent in the geological material. Heat treatment could impact fractionation by causing lighter isotopes to preferentially evaporate out of the ore, producing a negative shift in Δ199Hg values. Indeed, studies have demonstrated this anticipated enrichment in heavy Hg isotopes [60–62] and there is even some evidence for enrichments in both light and heavy Hg isotopes relative to unprocessed cinnabar ore [63]. However, it is unlikely that archaeological cinnabar was intentionally heat treated by ancient peoples to levels that would imitate the calcination effects of industrial mining, as the culturally desired visual properties of cinnabar (e.g., color) are incompatible with heat treatment.

While cinnabar (α-HgS) is stable under normal conditions, it will convert to metacinnabar (β-HgS), an isometric black mineral, at temperatures exceeding 260–315° C. This characteristic explains the lack of cinnabar-based slip painting in the Andes, and the preference for its post-fire application [36]. This characteristic also limits the potential impact of heat on mercury fractionation in the mineral since preparation of the pigment could not have reasonably exceeded such temperatures without risk of conversion to blackened metacinnabar and a loss of desired visual properties of the mineral. If heat treatment were part of the process to produce powdered cinnabar in ancient Andean contexts, heat levels would have to have been maintained below 300°C to avoid blackening, a material property that decreases the likelihood of thermally stimulated fractionation in archaeological pigments. Thus, we can assume that fractionation signatures of isotopes from processed cinnabar pigments should be consistent with those occurring in the natural ore source material. From this perspective, mercury isotope ratios detected in archaeological pigments offer significant potential to determine the ancient geological sources of cinnabar.

### Use of mercury isotopes for geochemical sourcing

The application of Multicollector Inductively Coupled Plasma Mass Spectrometry (MC-ICP-MS) in the analysis of mercury isotope systems has shown considerable promise to distinguishing among geological sources [64,65]. While all Hg isotopes are subject to mass dependent fractionation (MDF), ¹⁹⁹Hg and ²º¹Hg may also undergo mass independent fractionation (MIF) caused by magnetic isotope, nuclear spin, and nuclear volume effects [66–69]. The degree of MIF for these two odd-mass Hg isotopes adds another dimension to the commonly observed MDF, offering additional opportunity to distinguish between naturally occurring geological sources [61].

In the first geochemical study of stable mercury isotopes from archaeological cinnabar in the Andes, Cooke and colleagues [11] built a reference data set for Hg isotopes from the Huancavelica source (n = 5) and other known sources in Central and South America (n = 7) and compared their source database against 17 cinnabar samples taken from Peruvian artifacts that spanned a range of periods and geographical regions. They determined that the signatures of most of the artifacts were consistent with the Huancavelica deposit with a few notable exceptions. Samples from three Inka period coastal artifacts, referred to as “painted boards”, possessed elemental signatures that diverged from that of the Huancavelica ores, suggesting that an alternate cinnabar source was used on the south coast after 1400 CE. They concluded that the cinnabar deposit in the highland region of Huancavelica, Peru, may have served as the primary source of cinnabar for pre-Inka societies. Nonetheless, the limited sample size of the pilot study [11] left open the possibility that other, lesser-known cinnabar sources may have also been exploited.

Several dozen smaller cinnabar deposits have been recorded in Peru. The Hudson Institute of Mineralogy’s online data base (mindat.org) demonstrates a range of possible cinnabar sources, listing 2694 outcrops and mines across 66 countries, including five in Peru [24]. The explorations and descriptions of naturalist Antonio Raimondi [70] and geologist Georg Petersen [71] suggest many that small cinnabar sources occurred in various locations along the Andean mountains. Expanding upon Petersen’s [71] previous work, Brooks [72] presents a compilation of more than twenty cinnabar-mercury occurrences, offering a resource for assessing the regional availability of this important mineral in pre-contact Peru. Of the couple dozen known deposits, the Chonta and Queropalca occurrences were substantial enough to be exploited in the post-contact period in addition to the Santa Barbara Mine [71,72]. Controversies surrounding another potential cinnabar mine in Ecuador offer the possibility of additional Andean cinnabar sources outside of Peru [35,37,73]. As such, supplementary geochemical analyses have the potential to significantly alter current understandings of ancient Indigenous cinnabar exploitation and trade. The current study builds upon the work and methodology developed by previous studies [11,19,28] by enhancing the geochemical characterization of a pre-Hispanic ore source and contributing a substantial new dataset of additional archaeological specimens.

## Materials and methods

The Huancavelica mineralization belt is located in the highlands of Peru, covering an area measuring sixty kilometers long and three kilometers wide and oriented southeast to northwest under the modern city of Huancavelica. This is the largest source of cinnabar in the Americas, composed of forty-one cinnabar deposits, the largest of which is the Santa Barbara mine, concentrated in an area of around 90 meters wide and 550 meters long located near the summit [70,74]. This mineralization has been exploited from at least the 2nd millennium BCE into the 20^th^ century CE [19,29,71,75]. The collapsed Chaclatacana open pit, collapsed Colonial Period mine shafts, and historical architecture from mining operations dating from the late 16^th^ century through the 1970’s attest to an industrial level of exploitation, which has rendered the primary geological ore inaccessible. The Huancavelica mines are no longer actively exploited and there are very few extant geological samples with reliable provenience, save for five modern geological samples that were collected from unexhausted surface deposits and sampled by Colin Cooke from natural history museums. These five specimens formed the basis of the original characterization of the Huancavelica mineralization [11].

### Sampling

Robust characterization of the geological source is important for sourcing studies [63]; however, due to the massive size of the Huancavelica deposit, variations in ore formation processes across different veins of material may impact fractionation, producing a range of isotopic signatures throughout the ore body. This variability is compounded by the fact that ancient people may have collected cinnabar ore from part of the deposit not represented by the geological samples included in the study. Since pre-Hispanic mining pits have since been obliterated due to colonial and modern exploitation, the best record of the isotopic compositions of the veins that were actively exploited in the pre-Hispanic period are ancient archaeological samples that can be reliably traced to the Huancavelica deposit. For this reason, we define the Huancavelica ore source based both on geological samples and seven additional archaeological specimens excavated from the Atalla site.

Located in the Huancavelica region, a mere 13 km east of the Santa Barbara mine, the site of Atalla was home to residents who heavily exploited the Huancavelica source between 800 and 500 BCE [76–78]. As originally proposed by Burger and Matos [29], excavations at the site confirmed the economic and ritual importance of cinnabar pigment, which appeared frequently as a residue in stone and ceramic vessels and as a decorative pigment on ceramic and bone artifacts [77,78]. Because of Atalla’s proximity to the Santa Barbara mine area, we can confidently assume that the cinnabar pigment found in abundance in archaeological contexts originated from the Huancavelica ore deposit. The presence of Fe and Pb noted through portable X-Ray Fluorescence analyses carried out on the archaeological specimens of cinnabar from Atalla [77] support the notion that the Huancavelica deposit represents a secondary ore formation [79], as these elements are commonly associated elements in secondary formation contexts. Lighter red cinnabar, often formed by secondary sublimation, is purer and well suited for use as a pigment [79] which may account for the preferential exploitation of this source by pre-Hispanic peoples.

Seven (n = 7) cinnabar pigment residues excavated from the archaeological site of Atalla were aggregated with the original five (n = 5) geological samples collected by Colin Cooke and colleagues [11] to characterize the isotopic signature of the Huancavelica deposit. This improved the original isotopic characterization that was based solely on the geological source material by more than doubling the total number of specimens used to characterize the source. Furthermore, cinnabar sampled from Atalla should accurately reflect the isotopic signature of veins that were actually used in pre-Hispanic periods, and not just the isotopic signatures from outcrops that have persisted into the present. The inclusion of archaeological cinnabar into the definition of the geological signature thus ensures that ancient exploitation along now depleted veins is captured in our source characterization.

Building upon the reference samples and protocol previously developed by Cooke and colleagues, the current study brings together the results of 97 pigment specimens analyzed between 2012 and 2024, 65 of which have never been published. Of the previously unpublished specimens, 38 were recovered from well-documented archaeological excavations and 27 were sampled from objects in museum collections (S1 and S2 Tables). Additional information regarding the ethical, cultural, and scientific considerations specific to inclusivity in global research is included in the Supporting Information (S1 File).

Specimens were analyzed with either portable X-Ray Fluorescence, X-Ray Diffraction, SEM-EDS, or a combination of these methods to verify the presence of cinnabar in the sample prior to selection for follow-up spectrometric analyses. Periodization based on the Rowe-Menzel sequence [80,81] was used to facilitate temporal comparisons. For the purposes of this study, we define the periods as follows: Initial Period (1500–1200 BCE), late Initial Period (1200–800 BCE), Early Horizon (800–500 BCE), late Early Horizon (500–200 BCE), Early Intermediate Period (200 BCE–CE 600), Middle Horizon (600–1000 CE), Late Intermediate Period (1000–1400 CE), Late Horizon (1400–1532 CE), and Colonial Period (1532–1821 CE). Regional categorization and generalized periodization were employed to facilitate temporal and geographical comparisons of specimen groups, which were sourced from diverse contexts, some of which are separated by over 3000 years and hundreds of kilometers.

### MC-ICP-MS of Hg isotopes

Microsamples of cinnabar extracted from archaeological objects recovered in scientific excavations and housed in museum collections were analyzed at the Water Quality Center, Trent University. Cinnabar was digested in 5 mL of aqua regia and then sequentially diluted in 2% (v/v) HNO₃ to match the concentration of the bracketing Hg standard solution (2–5 ng/mL). A small fraction of the digest was used to measure the Hg concentration by cold-vapor atomic fluorescence spectroscopy (Tekran 2600, Tekran® Instruments Corporation, CA) following the U.S. EPA Method 1631. Ratios of stable mercury isotopes (¹⁹⁹Hg, ²ººHg, ²º¹Hg, and ²º²Hg) from these samples were measured using continuous-flow cold vapor generation coupled to MC-ICP-MS (Thermo-Finnigan Neptune or Nu Plasma II). Instrumental bias was corrected using the addition of an internal standard (NIST SRM 997 with ^205^Tl/^203^Tl ratio of 2.38714) and sample standard bracketing (SSB) with NIST SRM 3133 Hg standard solution. The analyses were performed at 1–3 ng g^-1^ Hg, and Hg concentrations of samples and standards were within 5% of each other. Following previous investigators [23,82], Hg isotope ratios are reported in standard delta notation (in permil units or %₀), relative to the NIST SRM-3133 Hg reference standard:


$$\begin{array}{c} \delta^{\text{x}}\text{Hg = }\left({{(}^{x}{Hg\text{/}}^{\text{198}}\text{Hg})}_{\text{sample}}\text{/}{{(}^{x}{Hg\text{/}}_{\text{198}}\text{Hg})}_{\text{std}}-1\right)\text{×1000,} \end{array}$$

where x = 199, 200, 201, 202, “std” is NIST SRM 3133 Hg. MIF of Hg isotopes was defined as the difference between the measured δ^x^Hg and the theoretically predicted δ^x^Hg based on the measured δ^202^Hg value [82], and expressed as:


$$\begin{array}{c} \Delta^{\text{x}}\text{Hg }\left(\text{‰}\right)\text{ = }\delta^{\text{x}}\text{Hg}-(\delta^{\text{202}}\text{Hg }\times \beta ), \end{array}$$

assigning β-values of 0.252, 0.502, 0.752, and 1.493 for Δ^199^Hg, Δ^200^Hg, Δ^201^Hg and Δ^204^Hg, respectively. Our repeated measurements of Hg isotope ratios for NIST SRM 8610 agreed with previous studies [82]. MDF is typically represented by δ^202^Hg, while MIF is characterized through ∆^199^Hg. Results are presented in S4 Table.

### IR-MS of S isotopes

Isotope Ratio Mass Spectrometry was also explored as a complementary method to MC-ICP-MS. Because this IR-MS was included as a “proof of concept” to explore the application of the technique, a limited number of specimens were selected to provide preliminary data that could be assessed for reliability and effectiveness. As such, four (n = 4) cinnabar specimens analyzed for Hg isotopes were also sent to the Fike Stable Isotope Biogeochemistry Lab at the Washington University at St. Louis where stable isotope analysis of S were carried out. Among these were two archaeological specimens from the site of Atalla, selected with the intent to characterize the source material from the Huancavelica ore deposit, and two additional specimens from pigments excavated from Chongoyape, with the hope of comparing S isotope results with the Hg isotope results captured through MC-ICP-MS.

For these analyses, approximately 400 µg sample (HgS) was loaded into tin capsules with excess V_2_O_5_. Samples with low purity (<50%) were weighed at ~1 mg and loaded with excess V_2_O_5_. The ^34^S/^32^S ratio was measured on a Thermo Delta V Plus, following combustion in a Costech ECS 4010 Elemental Analyzer. Sulfur isotope compositions are expressed in standard delta notation as permil (‰) deviations from the Vienna Canyon Diablo Troilite (VCDT) standard. Isotopic measurements were calibrated using 3 in-house standards that have been calibrated against international standards IAEA-S-1 (−0.3‰; [83]), IAEA-S-3 (−32.5‰; [84]), and NBS-127 (+21.1‰; [85]). Measurement uncertainty was monitored throughout the analyses using check standards with well-characterized isotopic compositions: Ag_2_S (0.3‰ ± 0.35), BaSO_4_ (+14.5‰ ± 0.33), and ZnS (−5.5‰ ± 0.55). Precision was determined to be ± 0.73‰ based on repeat analyses of check standards and sample replicates. Accuracy was determined to be ± 0.79‰ based on the difference between the observed and known values of the check standards and the long-term standard deviations of these check standards (after the methods in [86]). Results are presented in S3 Table.

### Statistical methods

Statistical methods were applied to the mercury isotope results to provide additional insight into the similarity of specimen groups with the signature of the Huancavelica source material. Ratios of Δ^199^Hg and δ^202^Hg from archaeological samples were plotted against the Huancavelica source, a group that was defined from a mix of twelve geological and archaeological specimens from the Huancavelica region. To facilitate statistical tests of the isotope ratio results, specimens were grouped by cultural and temporal affiliation based on provenience information and observed stylistic differences. These groups serve as statistical populations within which we might expect the same source to have been exploited. With support from the Indiana Statistical Consulting Center, multivariate statistical analyses in the form of a Hotelling’s T² test were performed on sample groups to test against the hypothesis that groups were distinct and not related to the Huancavelica source. To compare mercury isotope ratios of cinnabar specimen populations to the geological source, two different statistical tests were employed. The first test was formulated to determine if the cinnabar specimens originated from the Huancavelica ore source while the second test was formulated to compare across archaeological groups of interest.

All tests and models were run using R software version 4.4.1. Simple linear regression models and Mann-Whitney U tests were run using the stats package. Hotelling’s T² tests were run using the DescTools package [87]. For transparency and replicability, all data and code used in this paper have been posted to a GitHub repository: https://github.com/JustinMLin/Hg-isotope/

### Mann-Whitney U test on mercury isotope ratios

The available literature suggests that cinnabar specimens originating from the Huancavelica ore source exhibit a unique ratio of Δ^199^Hg and δ^202^Hg. This hypothesized ratio was estimated from the samples known to have originated from the Huancavelica ore source, then compared the ratios of the other samples to the estimated ratio.

To quantify the similarity of other samples to those from the Huancavelica ore source, distance to the Huancavelica regression line was used. These distances were calculated as follows:


$$d(x,y)=\;\frac{\left|\beta_{1}x-y+\;\beta_{0}\right|}{\sqrt{{\beta_{1}}^{2}+1}},$$

where

- x and y are the Δ^199^Hg and δ^202^Hg readings of a particular sample, and
- $\beta_{0}$ and $\beta_{1}$ are the intercept and slope of the regression line.

For each geological source, the distances from the Huancavelica line were computed and compared against the distances of the Huancavelica samples from the Huancavelica line. These two independent samples of distances were tested against one another using a one-sided Mann-Whitney U test at a significance level of α = 0.05. A one-sided test was employed to determine if the samples taken from the geological source were further away from the Huancavelica regression line than the Huancavelica samples. The non-parametric Mann-Whitney U test was employed, as opposed to a parametric test like the 2-sample t-test, because the samples of distances are not normally distributed. Thus, we deem groups with p values of <0.05 to be inconsistent with the Huancavelica source, as such a result suggests a less than 5% chance that the two groups derived from the same population (S5 Table).

### Hotelling’s T² test on mercury isotope readings

To compare the statistical similarity of the various specimen groupings, 2-dimensional Hotelling’s T² tests on Δ^199^Hg and δ^202^Hg values were used. These two-sided tests were run with a significance level of α = 0.05. In some pairings, the sample sizes were too small to adequately check test assumptions, so the test results should be interpreted with care. The test also did not apply to archaeological groupings with only one sample because the covariance structure cannot be estimated from one sample point.

## Results and discussion

### Huancavelica source characterization

A simple linear regression of the Δ^199^Hg against δ^202^Hg values of geological specimens produced a best fit line of y = −0.1394x − 0.1288 and R² value of 0.9913. The Atalla pigments were highly comparable to the characterization by Cooke and colleagues [11,19] but added an additional range of variation to create a best fit line of y = −0.1429x − 0.1189 with an R² value of 0.9515. While this line still indicates a strong fit, it also offers a more representative reflection of the natural variation inherent to the deposit of the ore body exploited in the pre-Hispanic period.

Values of Δ^199^Hg against δ^202^Hg from archaeological samples were measured with MC-ICP-MS then plotted against the Huancavelica source group. The consistency of Hg isotopic signatures from the geological ore specimens and the processed archaeological pigments supports our claim that pre-Hispanic populations were not processing cinnabar mineral for pigment in a way that significantly altered fractionation patterns. In order to facilitate statistical analyses, we grouped specimens into populations based on provenience as well as temporal and cultural affiliation in an attempt to produce more reliable statistical comparisons with the Huancavelica source. As predicted, most of the isotopic signatures for pre-Hispanic cinnabar are consistent with those recorded from the Santa Barbara mine area [11]; however, nine groups of pigment specimens presented p-values of less than 0.05 which suggest divergence from the Huancavelica source (S5 Table).

**Fig 2:**
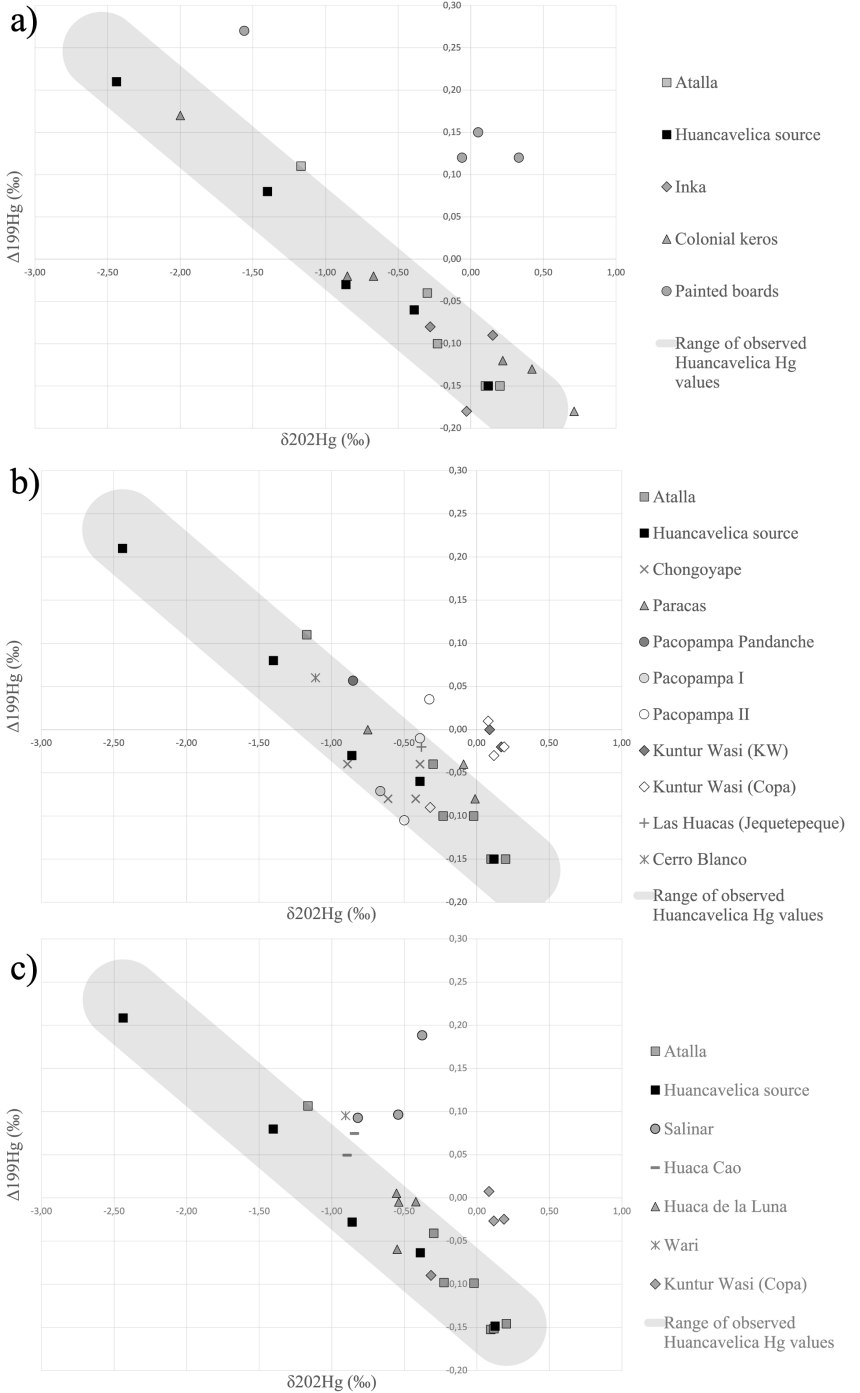
Hg ratios of samples groups plotted against Huancavelica source characterization. a) Cinnabar pigments found on Inka and Colonial Period artifacts fall within the range of variation of the Huancavelica source with the notable exception of the Painted boards group. b) Cinnabar pigments excavated from Initial Period and Early Horizon sites fall within the range of variation of the Huancavelica source with the notable exception of the Kuntur Wasi groups and one datapoint from Pacopampa II. c) Sample groups from the late Early Horizon through Middle Horizon. Salinar and Kuntur Wasi groups fall outside the range of variation of the Huancavelica source. The two Moche groups (Huaca Cao and Huaca de la Luna) form two distinctive clusters. Credit: Céline Erauw and Michelle Young.

**Fig 3:**
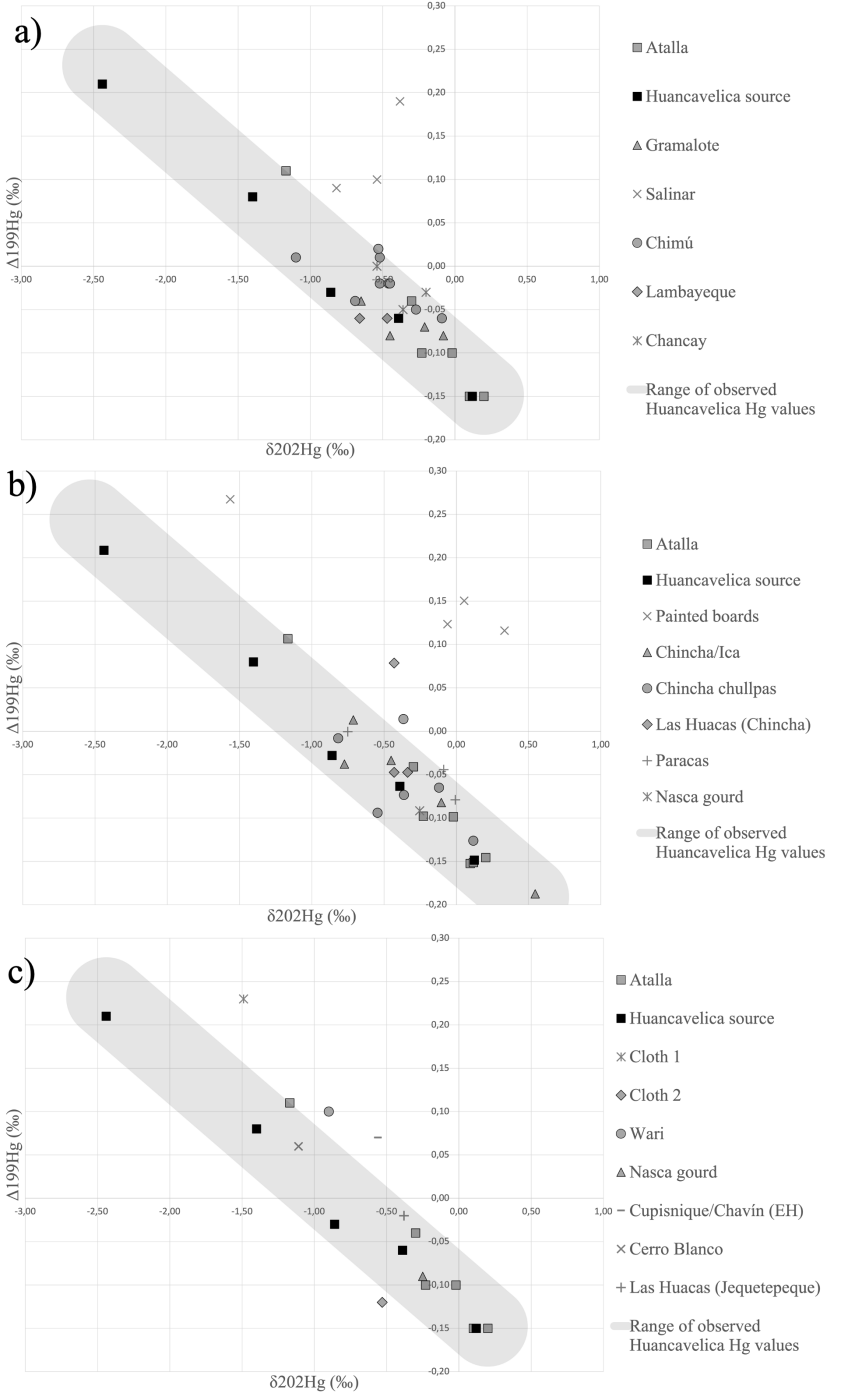
Hg isotope ratios of sample groups plotted against Huancavelica source characterization. a) Cinnabar pigments from the North and Central Coasts fall within the range of variation of the Huancavelica source with the notable exception of the Salinar group. b) Cinnabar pigments from the South Coast fall within the range of variation of the Huancavelica source with the notable exception of the Painted boards group and single datapoints from the Las Huacas (Chincha) and Chincha chullpas groups. c) Sample groups containing an isolated datapoint against Huancavelica source characterization. While the Nasca gourd and Las Huacas (Jequetepeque) points plot within the range of variation of the Huancavelica source, the other points do not. Credit: Céline Erauw and Michelle Young.

We also generated various forms of visualizations to support our interpretations of the results of statistical analyses. In addition to the p-values generated by the Mann-Whitney U tests, we utilized box-and-whisker plots to visualize overlap between sample groups and the Huancavelica source data based on the distance to the best fit line. Furthermore, we generated visualizations of the data through scatterplots (Figs 2 and 3). In these figures the orientation of the shaded band is defined by the linear regression of the Huancavelica ore group and its width is adjusted to encompass all Huancavelica ore samples. When comparative specimens’ values fall within this known range of Huancavelica ore values, we propose that specimens could have originated from the Huancavelica source.

### Comparing the Huancavelica source to archaeological sample groups from the Andes: temporal patterns

Our isotopic data suggest periods of continuity and disjuncture in the exploitation of cinnabar sources throughout Andean history. Mercury isotope ratios suggest continuity in the use of the Huancavelica ore source between the Initial and the Colonial Periods (1500 BCE–CE 1821) with a few notable exceptions (S5 Table). The Inka group consisted of one cinnabar specimen (n = 1) from lacquerer’s kit in the Goteborg Museum (1929.32.002) and two (n = 2) wood keros, ritual drinking vessels, held in the Museo Inka (230 and 231) while the Colonial keros group consisted of six (n = 6) carved Inka-Colonial keros, three of which are held in private collections and three of which are sourced from museum collections (A12, A13, and A14 in [11]). The Inka and Colonial keros cinnabar groups were consistent with the Huancavelica source and with each other, suggesting continuity in the source of cinnabar used for Inka and Inka-Colonial objects, particularly carved wood keros. On the other hand, painted boards from the Peruvian South Coast dating to the Late Horizon present an isotopic signature that differs significantly from the other Late Horizon and Colonial Period objects (see Fig 2a, S6 Table). We will discuss these objects in greater detail in the next section.

Conversely, specimens collected from contexts across Peru dating from the Initial Period through the Early Horizon (1500−500 BCE) offered examples of temporal shifts in cinnabar source exploitation. Pigment specimens from Pacopampa provided by Yuji Seki were divided into groups by period into Pacopampa Pandanche (1500−1200 BCE), Pacopampa I (1200−800 BCE), and Pacopampa II (800−500 BCE) based on the contexts from which these specimens were excavated. There are clear distinctions between the signatures of these groups that suggest shifts in the sources exploited during each period; perhaps at least two sources were used between the Initial and Early Horizon periods. Other sample groups from the same time span included in our sample consisted of four pigments excavated from Early Horizon burials at Chongoyape in Lambayeque Valley by Samuel Lothrop, a group of Paracas objects that include two post-fire painted Paracas vessels from the Fowler Museum at UCLA (X90.481 and X86.3814) and pigment from a bottle excavated from the site of Coyungo in the Nazca drainage, in addition to samples recovered from Cerro Blanco and Las Huacas (Jequetepeque) archaeological sites, located in the Nepeña and Middle Jequetepeque Valleys, respectively. While most of these pigments do not demonstrate a statistically significant difference from the Huancavelica ore source, the groups excavated from Early Horizon tombs at Kuntur Wasi in the upper Jequetepeque Valley diverge significantly (Fig 2b). This suggests that while most Initial period groups could have been acquiring cinnabar from Huancavelica, Kuntur Wasi residents relied on another, unknown source to furnish their burials. One specimen from the Kuntur Wasi (Copa) group does plot well the Huancavelica isotopic characterization, suggesting that at least some of the cinnabar used at Kuntur Wasi could have arrived from the Huancavelica region. Furthermore, one datapoint from Pacopampa II that falls outside of the range of Huancavelica source also appears to align along a linear regression with the main Kuntur Wasi group cluster, reinforcing the possibility that Huancavelica was one of two distinct cinnabar sources used in northern Peru during the Early Horizon.

In addition to providing the option to compare isotopic data against geological source data, our investigation also reveals spatial clustering which may offer another means of distinguishing analytically between exploited ore bodies. Cinnabar pigment excavated from two Moche sites, Huaca de la Luna and Huaca Cao Viejo, formed two Moche era (200–600 CE) sample groups. The ceremonial complex and adobe sculptural program from these two sites are ostensibly identical and employ similar recipes for producing pigments for mural art [88], suggesting close craft and ritual connections between these two centers. While both groups appear to fall along a linear distribution, they also demonstrate statistically significant clustering (S6 Table). Although the Hg isotope result clusters overlap with the range of variation present in the Huancavelica source (Fig 2c), both of these groups demonstrated low statistical correlation to the Huancavelica source (S5 Table). These clusters may correspond to different sources or to distinct exploitation areas of the Huancavelica ore deposit. Either way, this divergence suggests that, despite the remarkable correspondence in the ritual architecture and reliefs between Huaca Cao Viejo and Huaca de la Luna, the Moche peoples at each of these centers may have employed independent acquisition strategies to bring in foreign cinnabar from the distant highlands. Comparing isotopic sourcing data for imported materials such as cinnabar offers important insights into Moche political economy and the relationships between Moche centers, the complexity of which has also been underscored by recent research revealing the sacrifice of extralocal kin among Moche elite burials at Huaca Cao [89].

### Comparing the Huancavelica source to archaeological sample groups from the Andes: regional patterns

We compared groups originating from archaeological contexts on the Peruvian North Coast to understand how cinnabar exploitation changed within this region over time. The Gramalote group is composed of four samples excavated in Huanchaco from Initial Period contexts at the sites of Pampa Gramalote (Gramalote) (n = 3) and José Olaya–Iglesia Colonial (JO-IG) (n = 1). The Salinar group consists of three (n = 3) specimens excavated from late Early Horizon (La Iglesia phase) contexts at JO-IG in Huanchaco. The Chancay group is composed of three (n = 3) carved wood mummy masks from the NMAI that were previously analyzed (A10 and A11 from [11]) and one (n = 1) new specimen from the Walters Gallery (object 61.355). The Lambayeque group was composed of two (n = 2) specimens: a gold funerary mask from the Metropolitan Museum of Art (A9 from [11]) and a wood litter backrest decorated with copper alloy and wood figures in the Cleveland Museum of Art (CMA 1952.233). Finally, the Chimú group consisted of nine total pigments from sites in Huanchaco, two (n = 2) specimens excavated from Pampa La Cruz (PLC) and seven (n = 7) specimens excavated from the mass sacrifice site at Huanchaquito-Las Llamas (HLL) (Fig 1).

While the Hotelling’s T² Test revealed that the Chancay, Lambayeque, and Chimú groups were not statistically different from each other, the Salinar group was marked by a divergence in isotope ratios from Gramalote and Chimú groups (S6 Table). This difference is also supported by the Mann-Whitney U test which produced a p-value of 0.002198, indicating that the Salinar group originates from an unknown cinnabar source (Fig 3a). This finding suggests that peoples living in the Huanchaco area during the Initial Period may have acquired cinnabar from the Huancavelica source until the late Early Horizon, at which point the Salinar community (La Iglesia Phase) living at the site of JO-IG turned to new networks to acquire this prized, non-local mineral pigment. Such a shift in cinnabar exploitation suggests a radical reconfiguration of social and economic networks in the late Early Horizon (500−200 BCE), a period that aligns temporally with the disintegration of the Chavín Phenomenon and associated exchange networks [78,90].

Likewise, sample groups from the South Coast demonstrated consistency with the Huancavelica ore source and comparability with each other through the Hotelling’s T² Test. The Paracas group consisted of an early Paracas stirrup spouted bottle (X90.481) and a Middle Paracas spout and bridge bottle (X86.3814) from the Fowler Museum and pigment from the rim of a ceramic bottle spout excavated at Coyungo , a site located in the Nazca drainage. Also from the Nazca drainage, the Nasca gourd group included a single specimen from a gourd of unknown antiquity held in the National Museum of the American Indian (NMAI 199234). The Chincha/Ica group includes two (n = 2) carved wood posts from the Walters Gallery (61.351 and 61.352), and three hammered copper objects: two (n = 2) from the Cleveland Museum (CMA 1957.396 and CMA 1957.397) and one (n = 1) from the Harvard Peabody (42-28-30/4334). The Las Huacas (Chincha) group includes three (n = 3) specimens that date to the Late Horizon through Early Colonial Period excavated by Jordan Dalton from the site of Las Huacas, while the Chincha chullpas group is composed of six (n = 6) specimens excavated by Jacob Bongers from chullpas in the Middle Chincha Valley with evidence of use from the Late Intermediate to the Early Colonial Period. Despite the relative consistency of these sample groups, we note that the Las Huacas (Chincha) and Chincha chullpa groups each present a single outlier datapoint that is inconsistent with the Huancavelica source (Fig 3b), suggesting that the groups are not homogenous. These findings leave open the possibility that communities throughout the Chincha Valley used cinnabar ores that originate from more than one geological source.

The Painted boards group offers the most compelling case for a non-local signature of cinnabar. Our study added a new specimen to the three specimens (A15, A16 and A17) previously analyzed by Cooke and colleagues [11]. These “oar” like objects, which may have served as ceremonial digging tools, have been found in situ in high status tombs in the Ica and Chincha Valleys [36,80]. They have been radiocarbon dated to the Late Horizon [11], a period in which Inka influence facilitated the expansion of Chincha trade networks into Ecuador [91,92]. Thus, the Inka group shares chronological correspondence with the Painted boards and the Chincha chullpas group shares geographical continuity with the Painted boards; as such, these groups were selected for comparative pairwise testing. We found the Painted boards group to be inconsistent with the Huancavelica source and statistically incompatible with both the Inka and Chincha chullpas groups (S6 Table). The consistency of our new results with Cooke and colleagues’ [11] argument that painted boards from the Ica region were decorated with cinnabar pigment from an alternate geological source validates the accuracy of MC-ICP-MS as a method for distinguishing between geological sources. Our results offer further insights by demonstrating that at least two sources were used in the Inka period: one source that is isotopically consistent with the Huancavelica deposit was used to decorate Inka and Colonial Period keros, while another unidentified source was used on painted boards by people inhabiting the Ica Valley during the Inka period.

Although the role of the Chincha polity in long-distance exchange has yet to be fully clarified archaeologically, historical records describe the Chincha Valley as home to seafaring merchants [91,93–95]. The ethnohistorical data in tandem with our geochemical sourcing data from cinnabar used in Chincha elite burials during this period strongly suggest that long-distance merchants acquired cinnabar from an alternative source that they accessed through maritime networks. We can speculate that such networks may have reached to Ecuador, or perhaps beyond; however, future research is necessary to identify the geographical origin of this unknown source as well as to determine whether this exchange was driven by independent merchants or Inka administrative presence [92].

### Considerations and limitations in the use of MC-ICP-MS of Hg isotopes

One of the limiting factors in the use of mercury isotopes for geochemical sourcing of cinnabar pigments is the wide range of values observed from the Huancavelica source region. This variation may overlap with other geological sources whose isotope signatures also fall within that range. In some cases, our interpretations are also hampered by the paucity of specimens available for each analytical group. Cloth 1, Cloth 2, Cupisnique/Chavín (EH), Las Huacas (Jequetepeque), and Wari groups only contain one specimen each, making it difficult to attribute the results of these specimens to a particular source with confidence (Fig 3c). In these cases in particular, the p-value is not a reliable indicator of fit and we suggest further sampling and analyses to better define the Hg isotope signature of these groups. In the case of the Nasca gourd specimen, the Nasca region’s location in relative proximity to the Huancavelica highlands and the strong correlation with the Huancavelica characterization (p value = 0.9231) lead us to interpret it as a probable match with the Huancavelica source despite the small sample size.

Another complicating factor inherent to the analysis of archaeological materials is the possibility that human behavior has contributed to internal diversity of what we attempted to consider as a single population. A single archaeological site, context, artifact, or even pigment specimen may contain pigments sourced from more than one geological deposit, a reality that presents an unavoidable limitation to isotopic analysis of ancient colorants. In several cases, we attribute the low p-values reported through the Mann-Whitney U test to diversity present within our sample groups. For example, the Cupisnique/Chavín (IP) group is composed of six (n = 6) specimens that originate from objects across three museum collections: the Walters, Cleveland, and Fowler. These specimens were grouped together for our analysis based on stylistic similarities but do not necessarily share an origin of production, meaning that specimens in this group may not originate from the same geological source. Furthermore, we noted at least one outlier datapoint present across several of our groups including: Kuntur Wasi (Copa), Las Huacas (Chincha), and Chincha chullpas. The Las Huacas (Chincha) group spans several periods of use and in the case of the Chincha chullpas group, specimens spanned not only different periods but also different burial structures across a single valley. While we aimed to group specimens to increase the statistical accuracy of our analyses, we also acknowledge that some of our groups may not be sufficiently narrow – either culturally, politically, or temporally – to represent robust, normative populations that can be assessed through statistical means. Despite these limitations, our data offer statistically significant patterns of isotopic signatures that indicate different sources of cinnabar were exploited in the Andean region across three millennia.

### Sulfur isotope ratios

**Fig 4:**
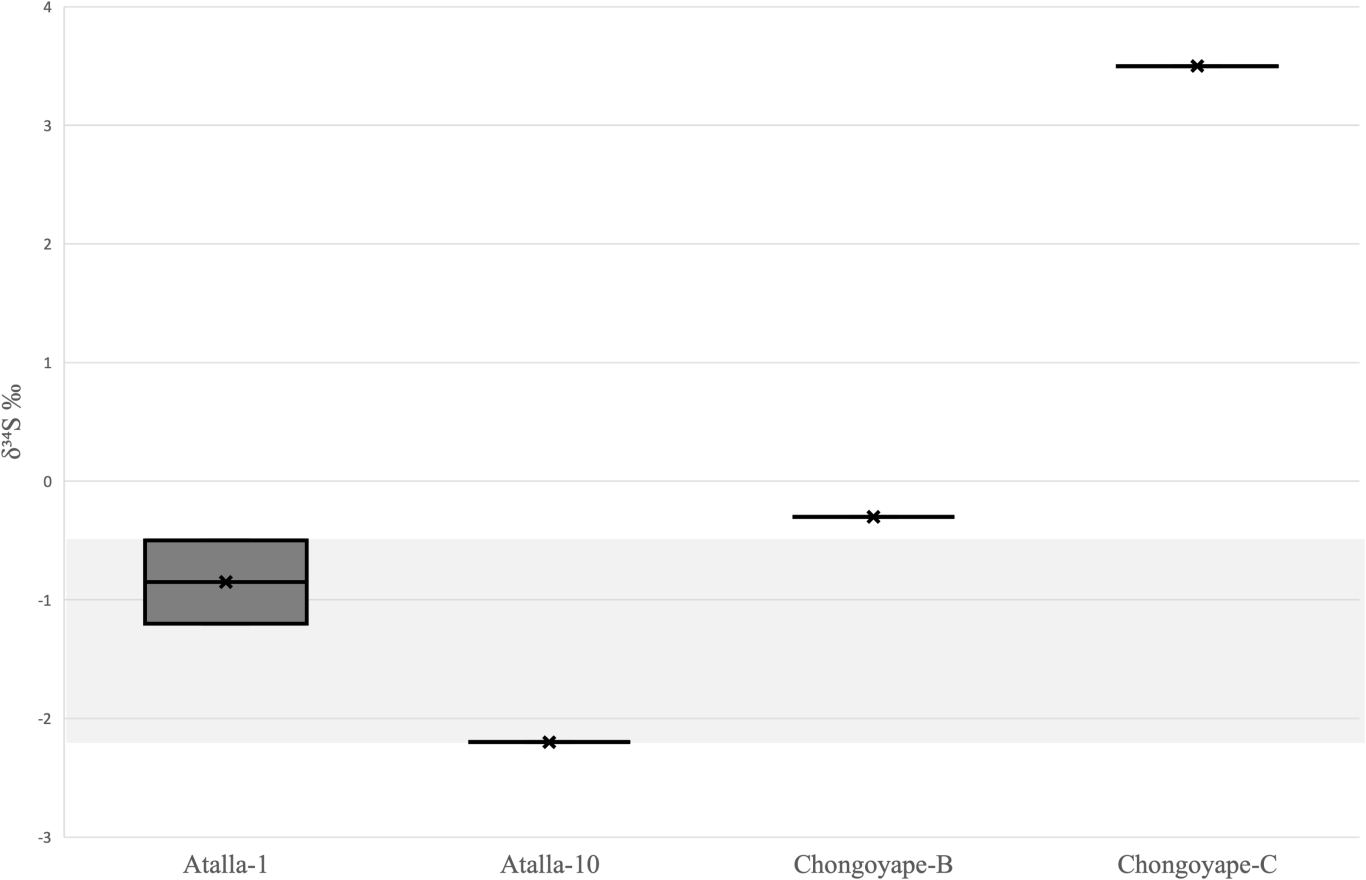
Box plot of δ^34^S measurements of cinnabar from Atalla and Chongoyape. Shaded band indicates range of Huancavelica ore source values for comparison with Chongoyape specimens. Credit: Céline Erauw and Michelle Young.

Often, the use of multiple isotope system enhances our ability to track sources of environmental samples. This prompted us to reconsider IR-MS as a method for sulfur isotope ratio determinations to assist with cinnabar sourcing. However, the δ^34^S readings from the Atalla pigments varied significantly, ranging between −0.5 and −2.2 ‰ (S4 Table). The most problematic observation for the replicability of this method was a lack of internal consistency for sulfur isotope ratios within the Atalla specimens. Multiple readings of the same sample material (Atalla-1) produced inconsistent results. Their averages were reported as −1.2 and −0.5 ‰, which were distinct from each other and did not overlap with the other Atalla cinnabar specimen, Atalla-10, within one standard deviation, despite the extremely high likelihood that these specimens both originated from the same geological deposit (Fig 4). Finally, these values did not overlap with those recorded for two of the Chongoyape pigments, despite these specimens being roughly comparable based on the mercury isotopes readings (Fig 2b). This variability calls into question the reliability of δ^34^S data for geochemical sourcing of cinnabar.

### Considerations and limitations in the use of IR-MS of S isotopes

Impurity of archaeological cinnabar presents a serious challenge for the use of sulfur isotopes in cinnabar sourcing. Pure cinnabar is 86.22% mercury and 13.78% sulfur; however, the true weight percent of sulfur recorded in each specimen deviated significantly from this value. The only relatively pure sample was Atalla-10, from the site of Atalla, while the average purity of cinnabar in the other samples was recorded between 13.2 and 54.0%. For archaeological pigments in particular, the high possibility of the inclusion of sulfur from sources beyond cinnabar presents a potential complication, since archaeological pigments often include other sulfur-bearing compounds. Past studies have shown that ancient craftspeople in the Andes and elsewhere in the Americas mixed cinnabar with other minerals such as hematite, gypsum, realgar, clays, and other substances [4,19,28,36,96]. Furthermore, pigment samples may also receive sulfur from organic sources. This may occur when artifacts are deposited in burials or could originate from intended or unintended additives to powdered pigments during the production process.

The ratio of Hg to S is a controlling factor in the stability of the color of cinnabar and an orange hue can develop with a surplus of sulfur and trace elements such as Al, K, and Si with corresponding amounts of Ca and Na to ensure stoichiometric equivalence [79]. The inclusion of trace elements may explain the neon orange to pink color of the cinnabar-based pigments identified in archaeological remains from Atalla [77]. However, the addition of lime or other additives threatens the purity of archaeological samples and could potentially affect the sulfur signatures of the cinnabar pigment. Alternate sources of sulfur such as gypsum (CaSO_4_) may also alter the S isotope values and create overlap between sulfur δ^34^S/δ^32^S ratios, both of which complicate interpretation of results [48]. Despite the ambiguity of the δ^34^S values, we believe in the importance of reporting these results to inform the work of future investigators. Adopting a multisampling strategy and improving protocols to ensure the consistency and purity of cinnabar samples could contribute to the reliability of IR-MS as a complementary geochemical sourcing technique for the future.

## Conclusions

The expense of MC-ICP-MS analyses, the sensitive nature of many collection objects, and the limited amount of pigment specimens available for any given analysis present challenges for geochemical sourcing in archaeological research. Nonetheless, the present study demonstrates the validity of Elisabetta Gliozzo’s [24] previous assessment that, although the total number of investigated cinnabar ores are few, it is still possible to obtain significant results through isotopic analyses. In carrying out such a study, we respond to Gliozzo’s call for caution to avoid incurring “false positives” [24]. By incorporating dozens of additional specimens, we have increased the available database used for the comparison of archaeological samples from the Andean region by more than threefold. This study of a larger total sample of cinnabar specimens (97 in total, 65 previously unpublished) demonstrates the promise of MC-ICP-MS for distinguishing between different political economies that resulted in divergent patterns of mineral acquisition in addition to determining probable geological sources of archaeological cinnabar.

Our results confirm the utility of mercury isotopes for geochemical sourcing. On the other hand, our exploratory foray into sulfur isotope ratios for cinnabar sourcing of demonstrates the need to proceed with caution due to low specimen purity and the high potential for sample contamination with other sources of sulfur in the environment, both of which are issues prevalent among archaeological materials in particular. Nonetheless, complementary isotopic data from sulfur isotopes and trace lead in the cinnabar ores may eventually offer additional analytical approaches to refine cinnabar sourcing; for example, Pb isotopes have been used with success for distinguishing between other kinds of materials such as ceramic glazes [97]. Furthermore, we want to underscore that the interpretive power of isotopic sourcing is greatly enhanced through multisampling rather than the use of isolated specimens to characterize the geochemical signature of cinnabar from a given site and period. Our results demonstrate the importance of establishing homogenous groups rather than single datapoints to reliably represent sites, periods or cultures, since we noted significant diversity, even within sample groups from the same site (such as the late pre-Hispanic to Colonial Period site of Las Huacas in Chincha) and among objects in museum collections from the same stylistic tradition (such as the Cupisnique/Chavín IP group).

Our results indicate that the Huancavelica deposit could have served as the primary source of cinnabar pigment for societies throughout much of Peru during the pre-Hispanic and Colonial Periods, beginning with exploitation by groups inhabiting Huanchaco on the North Coast of Peru during the Initial Period [4,19]. As the product of secondary formation and the largest source of cinnabar in the Americas, this abundant deposit possessed physical and visual characteristics that made it attractive to pre-Hispanic peoples for use as a powdered pigment over thousands of years. Our results also illuminate the presence of alternate cinnabar sources exploited by ancient communities in the Andes, including the residents of Kuntur Wasi in the Kuntur Wasi and Copa Phases of the Early Horizon, a Salinar group living at JO-IG in Huanchaco in the late Early Horizon, and groups who created painted boards in the Ica and Chincha Valleys during the Late Horizon. Although statistical analyses indicate that most of the other archaeological specimens included in this study were compatible with isotope ratios characteristic of the Huancavelica ore source, it is important to acknowledge that the lack of characterization of alternate sources limits our confidence of this assertion. As with any geological sourcing study, there always remains a possibility that an unknown and uncharacterized geological source may offer a stronger correlation with known archaeological specimens. Regardless, this study has produced a robust dataset of isotope ratios for archaeological cinnabar which can be assessed against any geological source material that may come to light in the future.

Though our understanding of the exploitation and trade of cinnabar in the Andes is still in its infancy, MC-ICP-MS analysis of mercury isotopes presents a promising avenue for continuing investigation. Understanding temporal and geographic patterns of the distribution of cinnabar pigment across Andean communities allows archaeologists to identify shifting exploitation strategies and craft production practices from pre-Hispanic times through the Colonial Period, contributing to a variety of archaeological discourses on economic exchange systems, mobility, technological innovation, long-distance sociopolitical relationships and the social and symbolic significance of different colorant materials. This study refines the isotopic signature of the Huancavelica cinnabar source, understood as one of, if not the primary source of cinnabar-based pigments in pre-Hispanic times. Beyond this, we have explored and validated a budding application for isotopic analyses for geochemical sourcing, which assuredly will have long-lasting implications for archaeology and other disciplines for decades to come.

## Supporting information

S1 Table Specimens incorporated in this study with associated provenance and chronological information presented in the groups used for statistical analyses. (XLSX)

S2 Table Museum information for specimens included in this study. (XLSX)

S3 Table Hg isotope data. (XLSX)

S4 Table Results from IR-MS analysis of pigment specimens. Note low percent purity of the samples and variation in the results for the same specimen.
(XLSX)

S5 Table Mann-Whitney U test results of specimen groups compared to Huancavelica characterization. (XLSX)

S6 Table Results of Hotelling’s T² Tests. (XLSX)

S1 File Inclusivity-in-global-research-questionnaire. (DOCX)
